# Co-morbidity of HIV, hepatitis B and syphilis among victims of sexual assault in the Transkei region, South Africa

**DOI:** 10.4102/phcfm.v1i1.54

**Published:** 2009-07-28

**Authors:** Banwari L. Meel

**Affiliations:** 1 Department of Forensic Medicine, Walter Sisulu University, South Africa

**Keywords:** sexual assault, human immunodeficiency virus (HIV), hepatitis antibodies, syphilis, rapid plasma regain antibodies

## Abstract

**Background:**

The human immunodeficiency virus (HIV), acquired immune deficiency syndrome (AIDS), hepatitis B and syphilis have a common mode of transmission, which is through sexual intercourse. These are also transmitted percutaneously and by blood transfusion. The purpose of this study was to determine the prevalence of HIV, hepatitis B and syphilis among victims of sexual assault by analysing serology results.

**Method:**

This is a record review of victims of sexual assault who attended the Sinawe Centre (a clinic for victims of sexual assault) between January and December 2004.

**Results:**

A total of 188 victims of sexual assault was reported. 35 (19.8%) tested HIV sero-positive. Hepatitis B antibodies were detected in seven (7.6%) and syphilis serology (RPR) was positive in five (2.9%). All were under 50 years of age, except one victim. Of the 35 who tested positive, 30 were below 30 years of age. Of those who were 30 years and younger, 12 were between 21 and 30 years old, 16 were between 11 and 20 years old and two were younger than 10. None was positive for all three tests. Two were positive for hepatitis B and HIV and two were positive for RPR and HIV.

**Conclusion:**

No significant co-morbidity of HIV, hepatitis B or syphilis was observed in this study, even though these diseases have the same mode of transmission.

## INTRODUCTION

Sexually transmitted infections (STIs) occur throughout the world; over 20 distinct pathogens are currently recognised. The World Health Organization (WHO) estimates that, worldwide, 250 million new STIs occur yearly. There is at least one new STI consultation for every 100 people per year in industrialised countries. Human immunodeficiency virus (HIV) boosts the virulence of STI pathogens,^1^ STIs being among the most common infections in the United States. Approximately 18.9 million new cases occurred in 2000, of which 9.1 million (48%) were among people aged between 15 and 24.^2^ A recent estimate in July 2008 showed that 5.7 million people living with HIV were in South Africa; approximately 3.2 million were women and 280 000 were children (aged between 0 and 14). HIV prevalence among women attending antenatal clinics was 29% in 2006 compared to 30.2% in 2005; among adults (aged between 15 and 49), HIV prevalence was 18.3% in 2006. Evidence points to a significant decline in HIV prevalence among young people (below 20 years of age), where prevalence was 13.7% in 2006 compared to 15.9% in 2005. There is significant variation in HIV prevalence by province, ranging from 39.1% in KwaZulu-Natal to 15.1% in the Western Cape. Inter-district HIV prevalence variation in the country is between 46% and 5.3%.^3^

STIs are now the most common group of notifiable infectious diseases in most countries, particularly in the age group of 15 to 50 years.^4^ The number of new STIs worldwide continues to rise annually. Many people, however, do not know that they are infected with an STI because it may remain asymptomatic until the disease reaches an advanced stage. Either up to half of STIs in women have no symptoms or infected women do not realise that their symptoms indicate a need to seek medical care.^5^

STIs are a major public health problem and can often lead to serious complications and sequelae, including infertility. Sub-Saharan Africa ranks first in STI annual incidence compared to other regions of the world. The WHO estimates that every year in Africa there are 3.5 million cases of syphilis, 15 million of chlamydial infection, 16 million of gonorrhoea and 30 million of trichomoniasis.^6^

Gonorrhoea, chlamydia, syphilis and chancroid are in the top 25 causes of healthy days of life lost in sub-Saharan Africa. In South Africa, an estimated 40% of women attending family-planning clinics were diagnosed with an STI; a total of 15% of women attending prenatal clinics in urban areas had latent syphilis. The World Bank estimates that over 3 million people in South Africa are infected with at least one STI per year; this means that one in ten sexually active people in South Africa may be infected with an STI every year.^7^ There is compelling evidence of the importance of STIs as a major determinant of HIV transmission. Approximately 11 million STI episodes are treated annually in South Africa, with approximately 5 million of these managed by private practitioners. Even without the HIV epidemic, STIs pose an important public health problem.^8^ Despite the availability of a preventative vaccine, however, hepatitis B remains one of the main sexually transmitted viral infections; approximately two-thirds of the total cases of hepatitis B are spread sexually.^9^ Based on serological estimates, a total of 1 250 000 prevalent cases of hepatitis B exists in the United States. Of these, approximately 750 000 are currently infectious people, their infection having been acquired sexually.^10^

The disease burden of classic STI has historically been heavy and continues to be a serious public health problem in South Africa. Morbidity from both ulcerative and non-ulcerative infections, particularly in women, is significant. The body of STI data, although mostly sound, remains incomplete, however, and, with the rapid emergence of HIV in South Africa, the surveillance of STIs and focused STI policies are critical.^11^ Victims of sexual assault face potential exposure during the assault but the risk of infection from a single assault is, in fact, lowA history of sexual abuse, however, increases exposure and thus the risk of contracting HIV.^12^ There is an increasing trend in sexual assault in this region of the Transkei because it is a poverty-stricken area; unemployment and incidental violence are very high. Women are responsible for raising their children and single parenting is common in this community. The sexual abuse of children is under-reported.^13^

The purpose of this study was to determine the co-morbidity of HIV, hepatitis B and syphilis among victims of sexual assault at the time of incident reporting.

## METHOD

The study was done at the Sinawe Centre, a clinic for victims of sexual assault attached to the Nelson Mandela Academic Hospital, Mthatha, Eastern Cape, South Africa. The Centre was established in 1999 and began functioning in 2000. It caters to a population of about 300 000 in the districts of Mthatha, Mquanduli, Ngqeleni, Libode, Tsolo and Engcobo. The female population in this region is assumed to be about half of the population. The clinic is a one-stop centre providing the multi-disciplinary management of victims of sexual assault only. This study was a record review of victims of sexual assault who attended the Centre between January and December 2004. Almost all the patients who visited the centre were female. The centre is open weekdays from 08:00 to 16:00. On weekends and after hours, victims were examined in the out-patients section of the Department of Gynaecology at the Nelson Mandela Academic Hospital and were followed up at the Sinawe Centre. It is a policy of the centre to provide HIV testing after counselling to all the victims. For this study, blood for HIV, hepatitis B and syphilis were taken with the consent of the victims. Post-exposure prophylaxis was offered to all the victims, except those who came later than 72 hours after the incident. Subsequent follow-up was advised after one week, four weeks, twelve weeks and six months. Initially, zidovudine and lamivudine were given for a week. HIV test results were available within 15 minutes and those who tested positive had an Elisa test done, which is a confirmatory test for HIV. The serology results were usually received within a week. All the victims were able to contact the doctor by phone any time during treatment. All the subjects’ files were reviewed systematically and results were calculated manually.

## RESULTS

**FIGURE 1:**
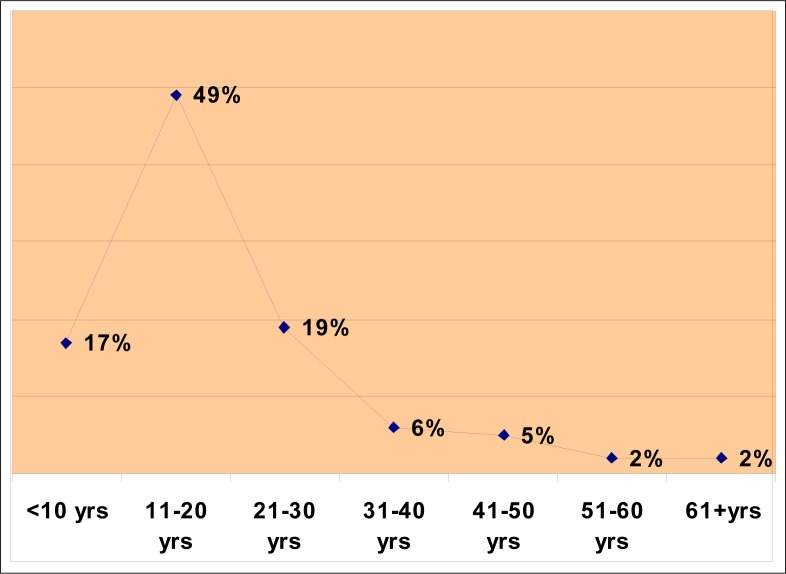
Percentages of different age groups of victims of sexual assault (n=188)

**FIGURE 2:**
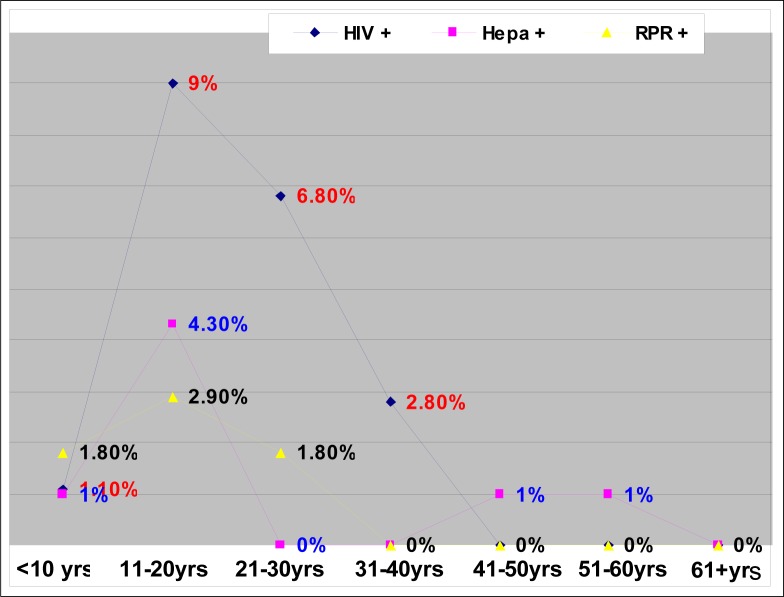
Relationship between HIV, hepatitis B and syphilis

A total of 188 victims of sexual assault was reported, of whom 35 (19.8%) tested HIV sero-positive. Hepatitis B antibodies were detected in seven (7.6%) and syphilis serology (RPR) was positive in five (2.9%). Except for one victim, all were under 50 years of age (Table 1 and Figure 1). Of the 35 who tested positive, 30 were below 30 years of age. Of those who were 30 years and younger, 12 were between 21 and 30 years old, 16 were between 11 and 20 years old and two were younger than 10. None was positive for all three tests. Two were positive for hepatitis B and HIV and two others were positive for RPR and HIV (Figure 2).

**TABLE 1:** Victims of sexual assaults in 2004 and seropositivity (n=188)

AGE GROUPS (YEARS)	VICTIMS OF SEXUAL ASSAUTS %	SEROLOGY TESTS (POSITIVE)		

		HIV (n=177)	HEPATITIS B (n=92)	RPR (n=171)
<10	32 (17%)	2 (1.1%)	1 (1%)	3 (1.8%)
11-20	92 (48.9%)	16 (9%)	4 (4.3%)	5 (2.9%)
21-30	36 (19.1%)	12 (6.8%)	0 (0%)	3 (1.8%)
31-40	12 (6.4%)	5 (2.8%)	0 (0%)	0%
41-50	9 (4.8%)	0%	1 (1%)	0%
51-60	3 (1.6%)	0%	1 (1%)	0%
61	4 (2.1%)	0%	0%	0%

**TOTAL**	**188**	**35 (19.8%)**	**7 (7.6%)**	**5 (2.9%)**

## DISCUSSION

This is the first study in this region of the Transkei based on serological results of STIs of victims of sexual assault. Transkei is a reservoir of mineworkers who are retired or were retrenched. It is estimated that 29.4% of all current mineworkers, who are mainly migrant labourers from all over Southern Africa, are HIV sero-positive.^14^ Miners in South Africa are now therefore more at risk of contracting STIs than becoming victims of mining accidents. The migrant-labour system is one of the underlying determinants in the transmission of HIV. The majority of mineworkers live alone, leaving their families at home, and are more vulnerable to contracting HIV. Other factors for the contracting of HIV are poverty, the practice of commercial sex, the low status of women, illiteracy, stigmatisation and discrimination.^15^ Some epidemiologists predict that mines could experience 12 000 to 40 000 deaths related to HIV/acquired immune deficiency syndrome (AIDS) by 2010.^16^ The serology results of the 188 patients who were tested at the Sinawe Centre were analysed and HIV was found in 19.8%, hepatitis B in 7.6% and syphilis in 2.9% (Table 1). Of the 188 who were screened, only 177 results for HIV, 92 for hepatitis B and 171 for syphilis were available (Table 1). In this study, one in five was HIV positive. This finding is similar to UNAIDS estimates for South Africa (one in five or six).^17^ A similar study done in Pretoria in 1999 showed an HIV prevalence of 19% and of 9% for hepatitis B.^18^ In 2003, the prevalence of HIV among young adults was estimated to be more than 38% in Swaziland, 37% in Botswana, 28.9% in Lesotho and 25% in Zimbabwe.^17^ The total number of people infected with HIV and the hepatitis B virus in South Africa was estimated from a number of sources of sero-prevalence. A total of 122 951 HIV-infected individuals was detected in South Africa in 1991. Of these individuals, 69% were from the urban black population and 20% were rural.^19^ The Transkei region is mainly rural, with low incomes and high unemployment.

STIs are among the most common illnesses in the developing world and have far-reaching health, social and economic consequences. The association between HIV and hepatitis B has been reported by numerous studies.^20^ It is therefore surprising that there is a significantly low infection of hepatitis B (7.6%) compared to HIV in this study. It is estimated that 45% of the world's population lives in areas where hepatitis B infection is high (8% or higher).^21^ The prevalence of hepatitis B and the co-infection of HIV in Thai patients, for example, is significant.^22^ This is because of the scanty reservoir of hepatitis B in the local community. This is an advantage because mandatory hepatitis B vaccination could be enormously beneficial in protecting the community. This would further contain the existing reservoir.

In this study, about half the victims (48.9%) were between 11 and 20 years old. This same group had the highest sero-prevalence of HIV (9%), hepatitis B (4.3%) and syphilis (2.9%) (Figure 2). RPR was negative in those over 30 years of age and HIV was negative in those over 40 years of age. Hepatitis B serology was positive in two individuals who were over 40 years of age (Table 1).

The 11 to 20-year age group is the school-going population (Figure 1). For the individuals of this group to be sero-positive for these three infections, they must have been sexually active from a very young age (Figure 2). This is alarming news for public health workers. 60%t of new HIV infections occur before the age of 25 years. The ‘Love Life’ campaign appropriately targets the country's 14 million 12 to 17 year-olds, more than half of whom have a greater chance of contracting HIV/AIDS.^23^ Both immediate and underlying factors contribute to HIV transmission in South Africa. Immediate determinants of the HIV/AIDS epidemic include behavioural factors, such as the frequency of unprotected sexual intercourse and multiple sexual partners, and biological factors, such as the high prevalence of STIs.^15^

HIV and other STIs remain highly associated in South Africa, which presents opportunities for the targeting of diagnostic and care interventions.^24^

South Africa has to learn from the rising incidence of HIV/ AIDS and more active measures need to be taken to prevent sexual assault. Despite the government spending enormous amounts of money on free condom distribution, there is no sign of the transmission of HIV declining. In 1990, for example, the prevalence of HIV/AIDS in both Thailand and South Africa was 0.7%. A decade later, the rate in Thailand had stabilised at less than 1.8% in the reproductive age group, whereas, in South Africa, it had risen to 20%.^25^ Syphilis is also still prevalent among young adults (younger than 30 years of age). A more aggressive approach to educating this group therefore needs to be adopted to contain this treatable disease. The limitation of this study is that it is a laboratory-based study and that it cannot differentiate hepatitis B antibodies due to previous vaccination or acquired infection. It does, however, provide an insight into the problem of co-morbidity of STI in victims of sexual assault.

No significant co-morbidity of HIV, hepatitis B or syphilis was observed in this study, even though these diseases have the same mode of transmission. There is a need for further study in a larger sample size.
